# Cathepsin B-Responsive Liposomes for Controlled Anticancer Drug Delivery in Hep G2 Cells

**DOI:** 10.3390/pharmaceutics12090876

**Published:** 2020-09-14

**Authors:** Seulgi Lee, Su Jeong Song, Jeil Lee, Tai Hwan Ha, Joon Sig Choi

**Affiliations:** 1 Department of Biochemistry, Chungnam National University, Daejeon 34134, Korea; smileleesg@cnu.ac.kr (S.L.); song.sj87@kiom.re.kr (S.J.S.); jeillee@o.cnu.ac.kr (J.L.); 2 BioNanotechnology Research Center, Korea Research Institute of Bioscience and Biotechnology (KRIBB), Daejeon 34141, Korea; 3 Department of Nanobiotechnology, KRIBB School of Biotechnology, Korea University of Science and Technology (UST), Daejeon 34113, Korea

**Keywords:** drug delivery, liposome, GLFG peptide, cathepsin B

## Abstract

In recent decades, several types of anticancer drugs that inhibit cancer cell growth and cause cell death have been developed for chemotherapeutic application. However, these agents are usually associated with side effects resulting from nonspecific delivery, which may induce cytotoxicity in healthy cells. To reduce the nonspecific delivery issue, nanoparticles have been successfully used for the delivery of anticancer drugs to specific target sites. In this study, a functional polymeric lipid, PEG-GLFG-K(C_16_)_2_ (PEG-GLFG, polyethylene glycol-Gly-Leu-Phe-Gly-Lys(C_16_)_2_), was synthesized to enable controlled anticancer drug delivery using cathepsin B enzyme-responsive liposomes. The liposomes composed of PEG-GLFG/DOTAP (1,2-dioleoyl-3-trimethylammonium-propane (chloride salt))/DPPC (dipalmitoylphosphatidylcholine)/cholesterol were prepared and characterized at various ratios. The GLFG liposomes formed were stable liposomes and were degraded when acted upon by cathepsin B enzyme. Doxorubicin (Dox) loaded GLFG liposomes (GLFG/Dox) were observed to exert an effective anticancer effect on Hep G2 cells in vitro and inhibit cancer cell proliferation in a zebrafish model.

## 1. Introduction

Cancer is a disease involving the abnormal proliferation of cells with deleterious effects on human health. Cancer arises from protein overexpression, and mutations in gene, which may inhibit cell growth and lead to metastasis. The latter results in the spread of cancer cells to distal organs, including liver, brain, breast, bone, and colon. Patients with cancer experience severe pain and are prone to developing complications. Currently, some of the most commonly administered cancer therapies include chemotherapy, radiation, and surgery, depending on the type of cancer. However, chemotherapy and radiation are associated with high levels of cytotoxicity and risk of injury to normal cells [1]. Conventional anticancer drugs such as doxorubicin, cisplatin, docetaxel, and gemcitabine, are also used to treat cancer by inhibiting the growth and proliferation of cancer cells [2,3,4]. These drugs can exert side effects as well, such as interference with DNA repair, DNA damage, and disruption of cellular membranes or proteins. Nanomedicines have been developed to overcome these challenges and increase the specificity of drug delivery to reduce the side effects of cancer therapy [5].

Nanomedicines are used for the diagnosis and treatment of several diseases. In recent times, various methods and materials have been developed for applications in nanomedicine. Depending on the aim of treatment, these include the characterization, designing of biocompatible and biodegradable materials, and formulation of nanoparticles, such as liposomes, dendrimers, micelles, polymeric nanoparticles, and inorganic nanoparticles. Nanoparticles can increase the water solubility of non-soluble drugs, protect genes from cellular enzymes, target specific cells, and regulate the release of therapeutic drugs. Owing to these properties, nanoparticles have the potential to be used in medical devices for enhanced diagnosis and treatment of diseases [6,7,8].

Liposomes are typical nanoparticles that can be constructed using various amphiphilic lipids and cholesterol. These spherical vesicles form a lipid bilayer structure owing to the self-assembling nature of the hydrophilic head groups and the hydrophobic parts in aqueous solution. Liposomes can be composed of various lipids, and the physicochemical properties of liposomes are dependent upon the lipid components and the drugs encapsulated [9,10]. Recently, the potential applications of liposomes have been investigated using genetic materials, functional peptides, magnetic materials, and proteins in several studies. Among the reported findings, aminopeptidase N was found to be overexpressed in tumors, while the asparagine-glycine-arginine (NGR) peptide was found to be capable of ligand-directed delivery [11]. This liposome has a modified pH-sensitive moiety, which enhances drug efficacy. In another study, a photosensitizer polymer was designed to form light-sensitive liposomes with surface-conjugated Her2 antibody. The Her2 receptor is known to be overexpressed in breast cancer cells. Compared to the control liposomes, the newly designed liposomes exhibited greater cytotoxicity and enhanced uptake efficiency [12]. The cancer environment is an important factor in the formulation of drug delivery carriers and can be used to increase drug delivery efficiency and regulate the release of anticancer drugs. Over the last decade, several types of liposomal anticancer drugs, such as Lipusu^®^ and Marqibo^®^ have been approved or have qualified at various stages of clinical trial for cancer therapy [13,14]. Recent studies on liposomal nanomedicine have been performed for studying multifunctional properties, such as targeted therapy, triggered activation, and enhanced permeability and retention (EPR) effect [7].

Cathepsin B is a lysosomal protease that plays important roles in the survival of cells, including autophagy, neuroprotection, and cell migration. It is overexpressed in several cancer cells types and is associated with cancer progression and metastasis [15]. As such, several studies have attempted to develop therapeutic strategies that target cancer cells expressing cathepsin B [16,17].

**Figure 1:**
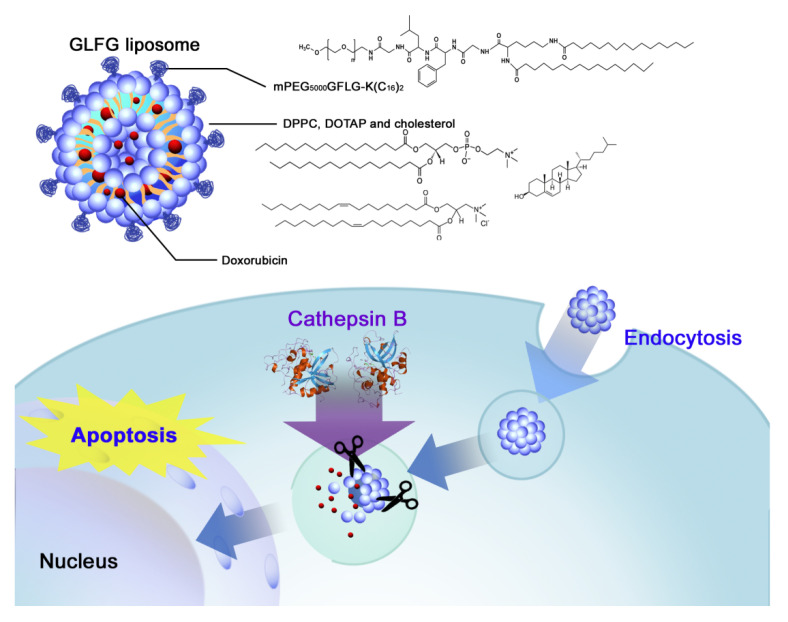
Schematic presentation of GLFG/Dox liposomes (doxorubicin loaded GLFG (Gly-Leu-Phe-Gly) liposomes) for enzyme-triggered drug delivery.

In this study, we designed a cathepsin B-responsive liposome using the ‘GLFG’ oligopeptide for the delivery of the anticancer drug, doxorubicin (Dox). A cathepsin B-cleavable peptide linker was introduced into the PEG lipid for the site-specific release of encapsulated drugs. The GLFG liposome was constructed using PEG-GLFG-K (C_16_)_2_ (PEG-GLFG), DPPC, DOTAP, and cholesterol [18]. As shown in Figure 1, the release of Dox by doxorubicin (Dox)-loaded GLFG liposomes (GLFG/Dox) was triggered upon treatment cathepsin B. GLFG/Dox could be introduced inside cells by endocytosis, and the lipids were hydrolyzed due to low pH and enzyme activation within the endosome/lysosome [19,20]. GLFG/Dox were evaluated based on their anticancer effects on Hep G2 cells in vitro and in a zebrafish model transplanted with Hep G2 cells [21].

## 2. Materials and Methods

### 2.1. Materials

DSPE-PEG (5000) amine, Dipalmitoylphosphatidylcholine (DPPC), 1,2-dioleoyl-3-trimethylammonium-propane (chloride salt) (DOTAP) were purchased from Avanti Polar Lipids (Birmingham, AL, USA). Palmitic acid and diethyl ether were obtained from Junsei chemical (Nihonbashi-honcho, Chuoku, Tokyo, Japan). Doxorubicin HCl was purchased from MedKoo Bioscience (Chapel Hill, NC, USA). Fmoc-Gly-OH, Fmoc-Phe-OH, Fmoc-Leu-OH, Fmoc-Lys(Fmoc)-OH and 2-(1H-benzotriazole-1-yl)-1,1,3,3-tetra-methyluronium hexaafluorophos -phate (HBTU) were purchased from Novabiochem (Darmstadt, Germany). Methxoypolyethylene glycol (PEG) amine (MW 5000) was purchased from NOF Corporation (Ebisu, Shibuya-ku, Tokyo, Japan). 1-hydroxybenzotriazole hydrate (HOBt) was obtained from Anaspec (Fremont, CA, USA). *N*,*N*-diisopropylethylamine (DIPEA), 4-(2-hydroxyethyl)-1-piperazine-1-ethanesulfonic acid (HEPES), piperidine, Cathepsin B from human liver, MTT ((3-(4,5-Dimethylthiazol-2-yl)- 2,5-Diphenyltetrazolium Bromide)), and dimethylformamide (DMF) were purchased from Sigma Aldrich (St. Louis, MO, USA). CellTracker™ Green CMFDA Dye was purchased from Thermo Fisher scientific (Carlsbad, CA, USA). Antibiotic-antimycotic (100×) solution, Dulbecco modified eagle medium (DMEM), Dulbecco phosphate-buffered saline (DPBS), trypsin- ethylenediaminetetraacetic acid (EDTA) solution (0.25%), and fetal bovine serum (FBS) were purchased from Gibco (Gaithersburg, MD, USA).

### 2.2. Synthesis PEG-GLFG-K(C_16_)_2_ (PEG-GLFG)

PEG-GLFG was synthesized following Fmoc chemistry [16]. PEG was dissolved in DMF solvent with Fmoc-Gly-OH (4 eq), HOBt (4 eq), HBTU (4 eq), DIPEA (8 eq) and reacted for 16 h at room temperature. PEG-Gly-Fmoc was precipitated and washed twice using cold diethyl ether. The product was dried using N_2_ gas. To remove a Fmoc group, it was dissolved in 30% piperidine solution and reacted for 1 h 30 min, and then PEG-Gly-NH_2_ was precipitated using cold diethyl ether. Then, the sequential amino acid conjugation and deprotection procedure was conducted for “Leu-Phe-Gly” using different amino acid monomers, respectively to synthesize PEG-GLFG-K(diFmoc). After deprotection of the Fmoc groups, palmitic acid (8 eq) was further conjugated with HOBt (8 eq), HBTU (8 eq), and DIPEA (16 eq) and reacted for 16 h at room temperature. Final product was precipitated and washed using cold diethyl ether and dialyzed using a dialysis membrane tubing (MWCO 3500) in distillated water. Finally, the product was freeze dried and analyzed using ^1^H-NMR and ^13^C-NMR (Fourier 600, Bruker, Billerica, MA, USA) in DMSO-d^6^. PEG and PEG-GLFG lipid was dissolved in MeOH. Each sample was mixed with a 2,5-Dihydroxybenzoic acid (DHB) matrix. Sample-matrix mixture was loaded on a sample plate and air dried. The molecular weight of each polymer was analyzed using MALDI-TOF MS spectroscopy (Voyager-TOF Mass Spectrometer, Applied Biosystems Inc., Foster city, CA, USA).

### 2.3. Formation of GLFG Liposomes and Doxorubicin (Dox) Encapsulation

GLFG liposomes were prepared using the thin film hydration method. PEG-GLFG, DOTAP, Cholesterol, and DPPC were prepared at various molar ratios as shown Table 1. All lipid mixtures were dissolved in EtOH/chloroform (*v*/*v*, 1:1) solvent, which was dried using N_2_ gas and under vacuum for 20 min. Lipid films were hydrated in 5 mM HEPES buffer and sonicated for 30 min. Dox encapsulated liposomes were prepared by the same procedure as mentioned above. Then, 2 mg lipids and 0.5 mg Dox were prepared in EtOH/chloroform solvent in a glass bottle, dried using N_2_ gas, and further dried under vacuum for 20 min. Thin film were hydrated in 5 mM HEPES and Dox solution. Dox/liposomes were purified using size exclusion chromatography (SEC) (NAP-25, GE Healthcare Co., Chicago, IL, USA). PBS buffer (pH 7.4) was used as a mobile phase at a flow rate of 1 mL/min. Each sample’s absorbance was measured at 480 nm. Drug encapsulation efficiency was calculated as the following Equation (1):

(1) $$\mathrm{Encapsulated efficiency}(\%)=\frac{\mathrm{Encapsulated amount of Dox}}{\mathrm{Total input amount of Dox}}\times 100$$

### 2.4. Zeta Potential and Dynamic Light Scattering (DLS)

GLFG liposomes were prepared at 2 mg/mL in a 5 mM HEPES buffer. DLS and zeta potential were measured using a Zetasizer Nano ZS instrument (Malvern, London, UK).

### 2.5. Field Emission-Scanning Electron Microscopy (FE-SEM)

Morphology of GLFG liposomes were imaged using FE-SEM. Sample was diluted in distilled water, dropped on a silicon wafer, and dried overnight at room temperature. Sample wafer was coated using platinum (Pt) and measured using FE-SEM (S-4800, Hitachi, Japan).

### 2.6. GLFG/Dox Liposomes Degradation Test by Cathepsin B

GLFG/Dox (20 μM Dox) liposomes were treated with or without 0.25 μM cathepsin B and incubated at 37 °C for 4 h. Samples were measured at 480/590 (ex/em) using a fluorescence spectrometer LS-45 (PerkinElmer, Cambridge, UK).

### 2.7. Cell Culture and Cytotoxicity Assay

Hep G2 cells (human) were maintained in a 3 °C incubator with 5% CO_2_. Cells were grown in a DMEM medium (DMEM 95%, FBS 5%, and 1% antibiotics) and subcultured using 0.25% trypsin-EDTA solution. The cytotoxicity of polymers and empty liposomes at various concentrations was performed by the MTT method. Cells were seeded in 96-well plates at a density of 10,000 cells/well. Then, each well was treated MTT solution (5 mg/mL in DPBS) and incubated for 4 h. Finally, supernatant was removed from each well and DMSO was added to dissolve the violet formazans. The absorbance was measured at 570 nm using a VERSAmax microplate reader (Molecular Devices, Sunnyvale, CA, USA). This method was also used to evaluate the liposomal drug effect. Free Dox and liposomes/Dox were treated in Hep G2 cells at 2.5 and 5 μM of Dox.

### 2.8. Confocal Microscopy

Hep G2 cells (20,000 cells/well) were seeded in a µ-Slide 8-well dish and incubated for 24 h. Free Dox, control liposomes/Dox, and GLFG/Dox were treated at the same concentration of Dox (final 5 μM). Cells were incubated for 4, 8, and 24 h, and washed twice using DPBS. Nuclei were dyed using Hoechst 33258 (0.014 µg/µL). Doxorubicin was also imaged using a Zeiss LSM 880 confocal laser microscope (Zeiss, Oberkochen, Germany).

### 2.9. Zebrafish In Vivo

Wild type zebrafishes were maintained in a 28 °C incubator and photoperiod of 10D/14L. All experiments were conducted according to approved guidelines and regulations of the Animal Ethics Committee of Chungnam National University (CNU00160). Zebrafish eggs were maintained in egg water (sea salt) at 28 °C. After 48 h of post fertilization (hpf), dechorionated eggs were prepared in a petri dish. Tricaine solution (0.04%) was treated for 1 min. Hep G2 cells were stained using a cell tracker (CellTracker™ Green CMFDA Dye, Invitrogen, Carlsbad, CA, USA) and injected in yolk using a micro injector (≈100 cells/larva). Then, zebrafish larvae were treated with free Dox and liposomes/Dox for 48 h. The fluorescence was measured using a Zeiss LSM 880 confocal laser microscope (Zeiss, Oberkochen, Germany).

## 3. Results and Discussion

### 3.1. Characterizations of PEG-GLFG Lipid

**Figure 2:**
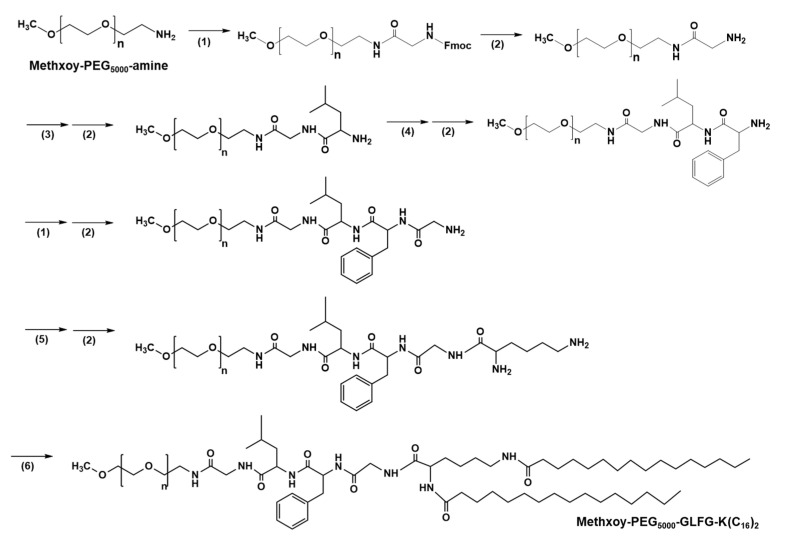
Synthesis scheme of PEG-GLFG. (1) HOBt (1-hydroxybenzotriazole hydrate), HBTU (2-(1H-benzotriazole-1-yl)-1,1,3,3-tetra-methyluronium hexaafluorophos -phate), DIPEA (*N*,*N*-diisopropylethylamine) and Fmoc-Gly-OH in DMF (dimethylformamide) for 16 h. (2) Piperidine 30% in DMF for 1 h 30 min. (3) HOBt, HBTU, DIPEA, and Fmoc-Leu-OH in DMF for 16 h. (4) HOBt, HBTU, DIPEA, and Fmoc-Phe-OH in DMF for 16 h. (5) HOBt, HBTU, DIPEA, and DiFmoc-Lysine-OH in DMF for 16 h. (6) HOBt, HBTU, DIPEA, and palmitic acid in DMF for 16 h.

**Figure 3:**
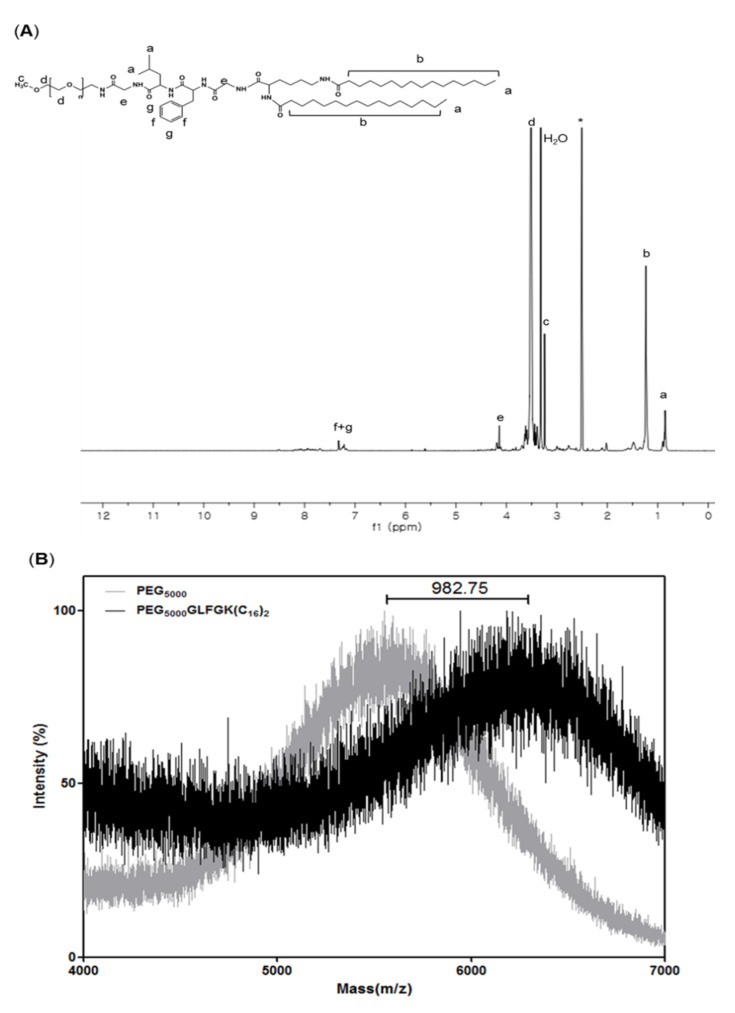
Measurements of PEG-GLFG using ^1^H-NMR and MALDI TOF mass spectroscopy. (**A**) ^1^H-NMR spectroscopy in DMSO-d^6^, (**B**) MALDI-TOF mass spectroscopy. 2,5-Dihydroxybenzoic acid (DHB) was used as a matrix.

PEG-GLFG lipid was synthesized using Fmoc chemistry as the synthesis procedure shown in Figure 2 [16]. PEG_-_GLFG was analyzed using ^1^H-NMR spectroscopy in a DMSO-d^6^ solvent. As shown in Figure 3A, PEG is at δ (in ppm) 3.25 [CH_3_OCH_2_–], 3.51 [–OCH_2_CH_2_–], glycine is at δ 4.14 [–COCH_2_NH–], leucine is at δ 0.86 [–CHCH_2_CH(CH_3_)_2_)], phenylalanine is at δ 7.23, 7.32 [–(C_6_H_5_)NH–], and palmitic acid signal is at δ 1.24 [–CH_2_CH_2_CH_2_–]. ^13^C-NMR of PEG-GLFG is provided in supporting information (Supplementary Materials Figure S1). The molecular weight (Mw) of native PEG and PEG-GLFG were measured by MALDI-TOF MS (Figure 3B). The Mw of native PEG was measured around 5549.1 *m*/*z*, and PEG_-_GLFG was about 6531.8 *m*/*z*. These result shows that the increase in Mw of 982.7 *m*/*z* is almost similar to the calculated value of GLFGK(C_16_)_2_ (979.5 *m*/*z*). Synthesis yield of PEG-GLFG lipids was 66.3%.

### 3.2. Characterization of GLFG Liposomes

GLFG liposomes consisted of four components, PEG-GLFG, DPPC, DOTAP, and cholesterol. PEG-DSPE was used to prepare the control liposomes. Several liposomes with varying quantities of PEG-GLFG were analyzed using DLS (Table 1). The GLFG 20 and GLFG 10 liposomes were 302.4 ± 4.2 and 334.8 ± 7.0 nm in diameter, while the GLFG 5 and GLFG 1 liposomes were 151.4 ± 1.6 and 151.7 ± 2.3 nm, respectively. The zeta potential values of the GLFG liposomes were determined as follows: GLFG 20: 2.7 ± 1.5 mV, GLFG 10: 21.0 ± 0.4 mV, GLFG 5: 25.2 ± 0.2 mV, and GLFG 1: 44.9 ± 1.9 mV. These results indicate that the zeta potential decreased at increasing levels of PEG. Owing to the PEG coating on the liposome surface, the charge on the liposome was affected from PEG density [22,23]. GLFG 1 had a higher positive surface charge value compared to PEG 20, which had a neutral zeta potential with a high PEG ratio. As indicated by the results, PEG can affect liposome surface charge owing to its shielding effect of PEG.

The GLFG liposomes were prepared using the thin layer method to encapsulate Dox. We performed the cytotoxicity effect of GLFG liposomes at various mole fractions with encapsulated Dox (final 2.5 μM) on Hep G2 cells and found that GLFG 5/Dox was more efficient compared to other compositions (Figure S2). Hence, GLFG 5 was selected for the characterization and further assessment of the following experiments. GLFG/Dox were separated from free Dox using SEC, and the mean size was measured using a Zetasizer Nano ZS instrument (280.3 ± 0.3 nm). The encapsulation efficiencies of GLFG/Dox (8.4%) and control liposomes (4.3%) were calculated.

**Figure 4:**
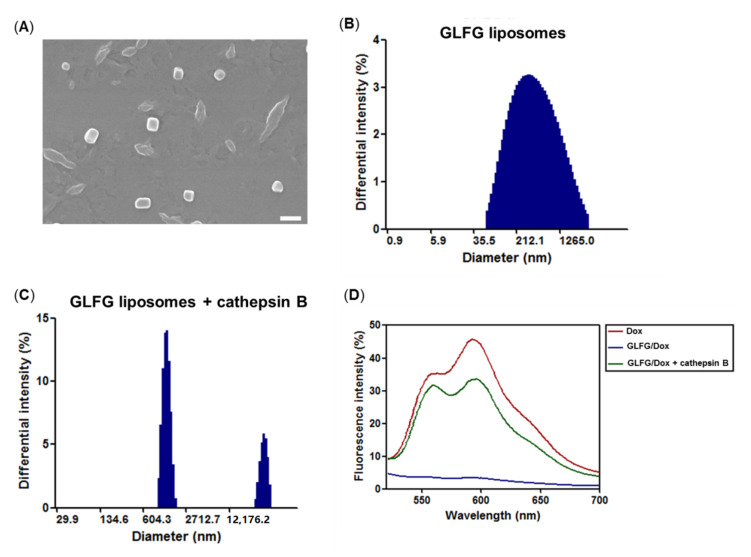
Characterization of GLFG liposomes and GLFG/Dox. (**A**) Morphology of GLFG liposomes by FE-SEM (scale bar = 200 nm), DLS of native GLFG liposomes (**B**), and GLFG liposomes treated with cathepsin B (**C**). (**D**) fluorescence spectroscopy of free Dox, GLFG/Dox, and GLFG/Dox treated with cathepsin B.

The morphology of the GLFG liposomes were imaged using FE-SEM (Figure 4A). FE-SEM images indicated a globular and uniform nano-sized structure of approximately 150 nm. The DLS results revealed a comparable size distribution and a similar diameter, which indicated that the GLFG liposomes formed a stable globular nanostructure. The characterization of GLFG/Dox was evaluated using cathepsin B treatment. Dox emits a fluorescence intensity a signal at 595 nm upon excitation at 470 nm (Figure 4D). Therefore, fluorescence intensity measurement could be used to confirm the release of the anticancer drug by cathepsin B. The untreated GLFG/Dox exhibited low fluorescence intensity, as Dox molecules were embedded in the liposomal membrane and the aqueous core of the liposomes. The fluorescence intensity of the Dox molecules within the liposome was quenched owing to molecular interactions with the lipids [24]. Cathepsin B-treated GLFG/Dox emitted a signal of higher fluorescence intensity [25]. Therefore, this result confirmed that cathepsin B triggered the release of Dox from GLFG/Dox. Additionally, the diameter of the cathepsin B-treated GLFG liposomes underwent changes (Figure 4B,C). The GLFG liposomes were stable at a size of approximately 200 nm. After incubation with cathepsin B, the size distribution of the GLFG liposomes increases by several micrometers. If the GLFG liposomes were degraded by cathepsin B, the stability would not have been maintained and the liposome structure would have collapsed. The balance between the hydrophilic PEG components and hydrophobic lipids in the liposomal structure would be lost, and the components would precipitate owing to aggregation in aqueous solution. As shown in Figure 4C, we confirmed that the mean diameter of GLFG liposomes increased significantly as indicated by DLS measurements. These results indicate that cathepsin B induced the collapse of GLFG/Dox and the subsequent release of encapsulated Dox. Cathepsin B is a lysosomal protease that is more active under acidic conditions. Therefore, the PEG-GLGF lipid of the liposomes were recognized and hydrolyzed by cathepsin B owing to the presence of enzyme-specific GLFG peptide sequence, which induced the collapse of GLGF/Dox and the subsequent release of Dox. The results indicate that the ‘GLFG’ sequence of PEG-GLFG was cleaved by cathepsin B, which helped regulate the controlled release of the drug targeting specific cancer cells [16,19].

### 3.3. Cytotoxicity Assay of GLFG Lipid and Liposomes in Hep G2 Cells

**Figure 5:**
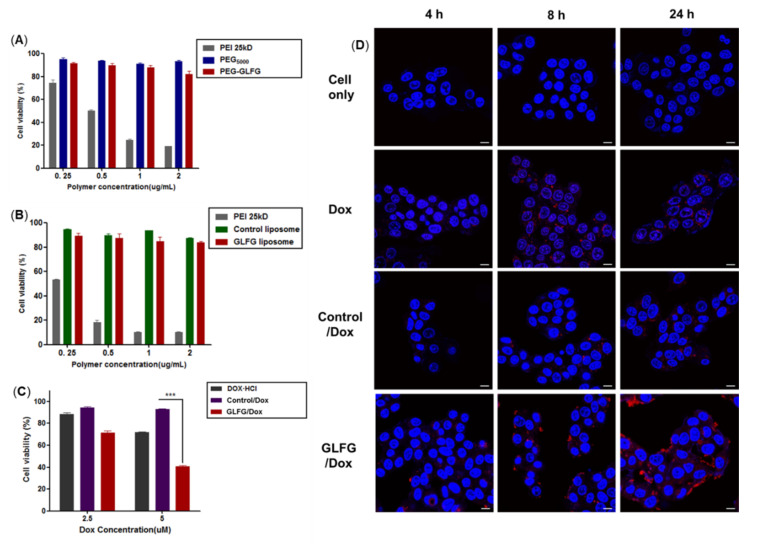
Evaluation of cytotoxicity for (**A**) PEG-GLFG and (**B**) GLFG liposomes. (**C**) Anticancer effects of GLFG/Dox in Hep G2 cells. (**D**) Cellular uptake of GLFG/Dox was observed using confocal microscopy. Nucleus (Blue, Hoechst33258), Dox (Red). The samples were treated using 5 μM of Dox. (scale bar: 10 μm)**.**

Cathepsin B is overexpressed in some of cancer cells. Several studies have reported that in infiltrative cancer, the cells secrete proteases, including cathepsin B, at high levels. Hep G2 cells exhibit high levels of cathepsin B activity [21]. The effects of PEG-GLFG and GLFG liposomes were studied in Hep G2 cells. To evaluate the cytotoxicity of the polymer, PEG-GLFG, the MTT assay was conducted. As shown in Figure 5A, PEI 25kD-treated cells showed 30% viability at treatment concentrations below 1.0 µg/µL, and PEI 25kD exhibited high cytotoxicity compared with PEG. Cells treated with PEG-GLFG exhibited over 80% viability at all concentrations, which could explain the negligible cytotoxicity. GLFG liposomes also exhibited over 80% viability on the cells (Figure 5B). These results indicate that PEG-GLFG and liposomes exhibited high biocompatibility in vitro with potential for use as drug carriers.

### 3.4. Anticancer Effect and Internalization of GLFG/Dox in Hep G2 Cells

As mentioned above, PEG-GLFG and GLFG liposomes exhibited low cytotoxicity in Hep G2 cells. The degradation of GLFG liposomes by cathepsin B was also confirmed in vitro. Next, to evaluate the anticancer effect in Hep G2 cells, samples were subjected to MTT assay (Figure 5C). Only free Dox found to cell viability (by 30%) at 5 µM. The control/Dox found to exert limited anticancer effects on Hep G2 cells. However, GLFG/Dox inhibited cell viability by 60%. In particular, GLFG/Dox exert a more pronounced inhibitory effect than control/Dox and free Dox [26,27]. These results revealed that GLFG/Dox can exert an effective anticancer effect owing to the enzyme-triggered controlled release of Dox based on the presence of the “GLFG” sequence. The internalization of GLFG/Dox in Hep G2 cells were confirmed using confocal microscopy. The cells were treated with 5 µM of free Dox, control/Dox, and GLFG/Dox for 4, 8, and 24 h. The sample groups were confirmed to show a low Dox intensity for 4 h. The cells treated with free Dox showed reasonable levels of fluorescence intensity at all times. However, the cells treated with GLFG/Dox observed a strong red signal for 24 h compared to those treated with free Dox or control/Dox. In particular, the GLFG/Dox treated group showed high fluorescence distribution in cells compared to the control/Dox treated groups. This demonstrated that the GLFG liposomes were enzymatically degraded by cathepsin B in the cells and released Dox upon the hydrolysis of the GLFG sequence of PEG-GLFG. The surface charge value of GLFG liposomes was higher than that of the control (Table 1). Therefore, the higher cationic charge on GLFG liposomes increased their intracellular uptake owing to enhanced interaction with the cell membrane. As a result, GLFG/Dox exhibited high cellular uptake and intracellular distribution. Therefore, it could be concluded that GLFG/Dox could deliver Dox efficiently at the target site and maintain its anticancer effect by facilitating the controlled release of encapsulated drugs.

### 3.5. Analysis of Anticancer Effects of GLFG/Dox Using a Cancer Injection Model in Zebrafish In Vivo

**Figure 6:**
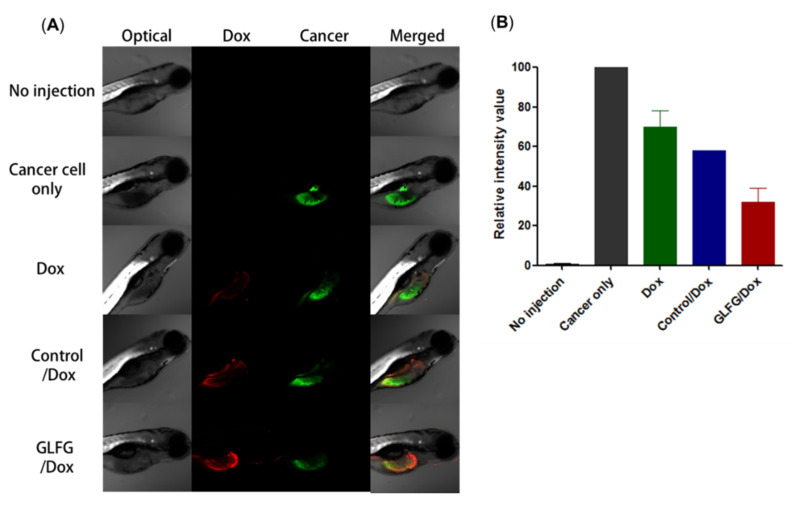
Anticancer assay in a Zebrafish model transplanted with labeled Hep G2 cells. Free Dox, control/Dox, and GLFG/Dox liposomes, each with 5μM of Dox, were used for treatment for 48 h, and (**A**) Dox (red) and labeled Hep G2 cells (green) were imaged using a confocal microscopy. (**B**) Quantification the fluorescence intensity of the cancer cells, (No injection (n = 3), cancer cells only (n = 1, two fish were dead), Dox (n = 3), control/Dox (n = 2, one fish was dead), GLFG/Dox (n = 3))**.**

A zebrafish cancer injection model was used to confirm the anticancer activity of GLFG/Dox (Figure 6). Hep G2 cells were dyed with a cell tracker (green) and injected into zebrafish yolk using a micro-injection system in vivo [28]. Free Dox, control/Dox, and GLFG/Dox were added to the larvae water at a concentration of 5 µM, and the larvae were incubated for 48 h. After incubation, the larvae were observed under a confocal microscope. The larvae treated with free Dox and control/Dox showed marginally lower cancer cell-associated fluorescence intensity than cancer cell-injected larvae. The larvae treated with GLFG/Dox exhibited low levels of cancer cell intensity, which indicates that GLFG/Dox caused significant inhibition effects on the transplanted cancer cells. These results demonstrated that cancer cell growth was inhibited to a higher degree in larvae treated with GLFG/Dox than that in the control group larvae [28]. This confirms that PEG-GLFG facilitated the circulation of GLFG/Dox in the bloodstream of zebrafish, and subsequently, increased the retention time of drugs and cancer-specific drug release, and thereby effectively exerted an anticancer effect. Therefore, GLFG/Dox represents a promising drug delivery system for use in cancer therapy.

## 4. Conclusions

In this study, a PEG-GLFG was designed with an enzyme-responsive “GLFG” sequence, and its synthesis was confirmed in ^1^H-NMR, ^13^C-NMR and, MALDI-TOF MS analysis. The PEG-GLFG and liposomes exerted negligible cytotoxicity in Hep G2 cells. GLFG/Dox were found to encapsulate Dox efficiently and form stable nano-sized liposomes, as confirmed in DLS and FE-SEM analyses. The effectiveness of GLFG/Dox was investigated in Hep G2 cells. GLFG/Dox were found to be sensitive to degradation by cathepsin B and exerted a more pronounced anticancer effect in Hep G2 cells and in a cancer cells-injected zebrafish model in vivo. Our results reveal the potential of GLFG liposomes as a novel drug carrier for cancer treatment. These findings suggest that PEG lipids containing functional peptides exhibit significant clinical potential in cancer chemotherapy.

## Figures and Tables

**Table 1:** Characterization of GLFG liposomes.

Liposome	PEG-GLFG:DPPC:DOTAP:Chol (Molar Ratio)	Size (nm) ^a^	PDI ^a^	Zeta Potential (mV) ^a^
Control	10:70:10:10	199.5 ± 8.7	0.3	7.2 ± 0.3
GLFG 1	1:79:10:10	151.7 ± 2.3	0.3	44.9 ± 1.9
GLFG 5	5:75:10:10	151.4 ± 1.6	0.3	25.2 ± 0.2
GLFG 10	10:70:10:10	334.8 ± 7.0	0.3	21.0 ± 0.4
GLFG 20	20:60:10:10	302.4 ± 4.2	0.3	2.7 ± 1.5

^a^ All measurements were repeated three times. (PDI: polydispersity index, PEG-GLFG, polyethylene glycol-Gly-Leu-Phe-Gly-Lys(C_16_)_2_, DPPC-dipalmitoylphosphatidylcholine, DOTAP-1,2-dioleoyl-3-trimethylammonium-propane (chloride salt)).

## Supplementary Materials

The following are available online at https://www.mdpi.com/1999-4923/12/9/876/s1: Figure S1: ^13^C-NMR spectrum of PEG-GLFG in DMSO-d^6^ (600MHz), Figure S2: The cytotoxicity effect of GLFG liposomes at various compositions with encapsulated Dox (final 2.5 µM) on Hep G2 cells.
