# Supplementary material for: Cathepsin B-Responsive Liposomes for Controlled Anticancer Drug Delivery in Hep G2 Cells
Source: Pharmaceutics. 2020 Sep 14;12(9):876. doi: 10.3390/pharmaceutics12090876 (PMC7558574; doi:10.3390/pharmaceutics12090876)
Supplement: Supplementary file 1 [file pharmaceutics-12-00876-s001.pdf]

pharmaceutics

MDPI

Supplementary Materials: Cathepsin B-Responsive Liposomes for Controlled Anticancer Drug Delivery in Hep G2 Cells

Seulgi Lee, Su Jeong Song, Jeil Lee, Tai Hwan Ha and Joon Sig Choi

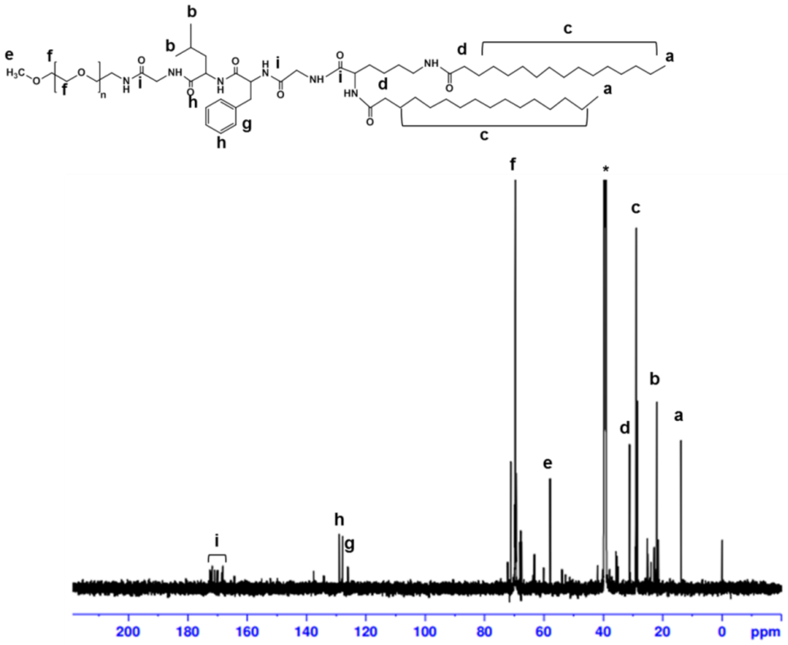

Figure S1. ^{13}C-NMR spectrum of PEG-GLFG in DMSO-d^{6} (600MHz).

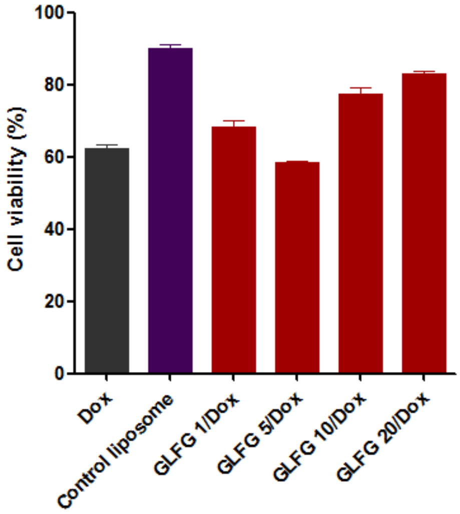

Figure S2. The cytotoxicity effect of GLFG liposomes at various compositions with encapsulated Dox (final 2.5 uM) on Hep G2 cells.
